# 2326 Prognostic value of left ventricular mitral annular longitudinal displacement measured by tissue Doppler imaging in patients with acute coronary syndrome

**DOI:** 10.1017/cts.2018.178

**Published:** 2018-11-21

**Authors:** Mats Lassen, Kristoffer G. Skaarup, Allan Z. Iversen, Peter G. Jørgensen, Flemming J. Olsen, Jan S. Jensen, Tor Biering-Sørensen

**Affiliations:** 1 University of California, San Francisco, CA, USA; 2 Copenhagen University Hospital Gentofte

## Abstract

OBJECTIVES/SPECIFIC AIMS: To investigate the prognostic value of left ventricular mitral annular longitudinal displacement (LD) measured with color tissue Doppler imaging (TDI) in a large population suffering from acute coronary syndrome (ACS). METHODS/STUDY POPULATION: In total, 501 ACS patients underwent an echocardiography within 9 days after a percutaneous coronary intervention. Regional LD was obtained from the 6 mitral annular regions with TDI and GLD was calculated as an average. RESULTS/ANTICIPATED RESULTS: During a median follow-up time of 4.4 years 46 ACS patients suffered CVD. Mean value of GLD in the population was 8.11mm (±2.4). GLD and LD obtained from the inferior wall remained significant independent predictors after multivariate adjustment for clinical parameters, GLD (HR: 1.43, 95% CI: 1.12–1.82, *p*=0.014, per 1mm decrease), inferior LD (HR: 1.38, 95% CI: 1.14–1.66, *p*=0.001). Furthermore, inferior wall LD was the primary source of prognostic information in GLD since only inferior LD remained significant when both measures were included in the same model: GLD (HR: 0.95, 95% CI: 0.64–1.40, *p*=0.781); inferior LD (HR: 1.60, 95% CI: 1.15–2.22, *p*=0.005). Of all walls, only inferior wall LD remained as an independent predictor after multivariate adjustment. DISCUSSION/SIGNIFICANCE OF IMPACT: GLD provides independent prognostic information in ACS patients over and beyond all conventional echocardiographic measures. Regional inferior LD was the primary source of prognostic information gained from GLD. GLD proved to be a better predictor of cardiovascular events than conventional echocardiographic measures. This could lead to better risk stratification in the clinical setting and open up for earlier intervention in high-risk individuals.

